# Advanced, recurrent, and persistent cervical cancer management: in the era of immunotherapy

**DOI:** 10.3389/fonc.2024.1392639

**Published:** 2024-08-05

**Authors:** Tatiana Galicia-Carmona, Eder Alexandro Arango-Bravo, Jaime A. Coronel-Martínez, Lucely Cetina-Pérez, Elva G. Vanoye-Carlo, Ricardo Villalobos-Valencia, José A. García-Pacheco, Patricia Cortés-Esteban

**Affiliations:** ^1^ Department of Clinical Research, Instituto Nacional de Cancerología (INCan), Mexico City, Mexico; ^2^ Department of Medical Oncology, Instituto Nacional de Cancerología (INCan), Mexico City, Mexico; ^3^ Department of Surgical Oncology, Hospital Regional de Alta Especialidad de la Península de Yucatán, Yucatán, Mexico; ^4^ Department of Medical Oncology, Centro Médico Nacional Siglo XXI, Instituto Mexicano del Seguro Social, Mexico City, Mexico; ^5^ NSR III Researcher assistant, Consejo Nacional de Humanidades, Ciencias y Tecnologías(CONACYT), Mexico City, Mexico; ^6^ Department of Medical Oncology, Centro Médico Nacional 20 de Noviembre, Mexico City, Mexico

**Keywords:** cervical cancer, metastatic cervical cancer, immunotherapy, recurrent and persistent disease, checkpoint inhibitors

## Abstract

Cervical cancer constitutes a significant health burden for women worldwide despite being preventable by vaccination and screening. Advanced stages of the disease are associated with a poor prognosis, and treatment approaches have seen little change over several decades, resulting in an overall survival rate of no more than 17 months. Additionally, there are limited options for second-line treatment. The urgent need for innovative and effective therapies to improve the outlook for this group of patients, along with an enhanced understanding of the interactions between the disease and the host’s immune system, has propelled immunotherapy into a rapidly advancing field with notable achievements. Among various immunotherapeutic approaches, immune checkpoint inhibitors emerge as the most advanced treatment option. Clinical trials assessing these inhibitors as single agents or in combination with chemotherapy show promising results. As immunotherapy begins to redefine standards of care for metastatic, recurrent, or persistent cervical cancer, this review addresses recent advances and current recommendations for its management in both first and second-line treatment. The goal is to provide insights into the evolving landscape of cervical cancer treatment, specifically focusing on immunotherapeutic interventions.

## Introduction

Despite being a completely preventable disease, in developing countries, cervical cancer (CC) is a major contributor to cancer-related deaths in women. For a long time, cisplatin as monotherapy or in combination represented the standard treatment for this patient group, with an overall survival not exceeding 13 months. In recent years, the advent of targeted therapies such as immunotherapy has significantly improved the prognosis for these patients.

## Immunity and cervical cancer

Human Papillomavirus (HPV) is a necessary but not sufficient etiological factor for developing cervical cancer. Infected basal epithelial cells hosting HPV express only early genes. However, HPV integration into the host genome leads to the expression of oncogenes E6 and E7 (1, 2). Integration of the HPV genome is a critical step in the development of HPV-associated cancers. This integration event preferentially occurs at fragile sites within the human DNA, regions characterized by increased susceptibility to breakage and rearrangement. The subsequent expression of viral oncogenes E6 and E7 is not only essential for the initiation and progression of premalignant lesions but also actively promotes genomic instability, further contributing to cellular transformation and malignant progression (3).

HPV-infected cells can evade immunosurveillance by inhibiting acute inflammation and immunological recognition. This viral and inflammatory cancer environment has been shown to be responsible for inducing PD-L1 expression (4). There is evidence that PD-L1 expression plays a significant role in creating an “immune privileged” site for initiating and persisting HPV infection by downregulating T-cell activity and generating adaptive immune resistance (5, 6). High-level PD-L1 expression is rare in healthy cervical tissue, but is increased in T cells and tumoral cells in 35 to 96% of cervical cancers (7).

Immunological escape is associated with local negative regulation as well as evasion of immune system detection, including increased regulatory T cells (Treg), loss of major histocompatibility complex (MHC) antigen presentation, chronic inflammation, and regulation of immune checkpoint molecules (8). Targeting tumor-specific antigens remains a cornerstone of cancer immunotherapy. However, the immunosuppressive tumor microenvironment presents a significant challenge, often hindering the efficacy of such targeted approaches. Therefore, therapeutic strategies aimed at reversing this immunosuppression within the tumor microenvironment are crucial for enhancing the efficacy of cancer immunotherapy. This can involve approaches such as inhibiting checkpoint molecules, depleting regulatory T cells, or promoting the activity of immunostimulatory cells and cytokines. Therapeutic interventions, such as immune checkpoint blockade targeting molecules like PD-1/PD-L1 and CTLA-4, aim to overcome this challenge; they are not specific for HPV antigens, and if successful, they can be efficient in the majority of cervical cancer cases, regardless of the associated HPV type (9). Tumor cells often exploit immune checkpoint pathways as a mechanism for immune evasion. Therapeutic interventions targeting immune checkpoints, such as PD-1/PD-L1 blockade, can restore T cell function and promote tumor cell killing. By preventing the inhibitory signals mediated by these checkpoints, T cell proliferation and cytotoxic activity against cancer cells are enhanced within the tumor microenvironment.

## Immune checkpoint inhibitors

The application of immunotherapy in CC treatment is grounded in several key molecular features observed in these tumors. Elevated Tumor Mutational Burden, Microsatellite Instability, high PD-L1 expression, and elevated Tumor Inflammatory State, suggest an environment conducive to successful immunotherapy intervention. Therefore, the convergence of these molecular features in CC provides a strong rationale for employing immunotherapy as a treatment strategy (10).

Various tumors, including CC, express PD-L1, an immune checkpoint molecule mediating tumor cell escape from immune system-mediated destruction (11). PD-L1 expression by tumors enables them to evade destruction by CD8+ T cells. PD-1 is a crucial immune checkpoint molecule involved in maintaining self-tolerance and modulating the immune response. During an inflammatory response to infection, PD-1 expression on activated effector T cells helps prevent autoimmunity by attenuating T cell activation. However, within the tumor microenvironment, PD-1 can contribute to immune resistance. PD-1 is expressed on various immune cells, including activated T cells and regulatory T cells (Tregs). Notably, PD-1 expression on Tregs, coupled with ligand engagement, enhances their proliferation and amplifies their immunosuppressive function. Furthermore, PD-1 expression extends beyond T lymphocytes to include B cells and other immune subsets (9).

PD-L1 and PD-L2 serve as the two ligands for PD-1. Binding of either ligand to PD-1 triggers a co-inhibitory signal within activated T cells, leading to suppression of their effector functions. In the context of cancer, PD-1 is frequently upregulated on tumor-infiltrating lymphocytes (TILs) across diverse tumor types (12). This, coupled with the common overexpression of PD-L1 on tumor cells, facilitates immune evasion by inhibiting anti-tumor T cell responses (13, 14). Further, PD-L1 expression is an independent prognostic factor for poor outcome, irrespective of established clinicopathological features, including stage, tumor size, depth of invasion, lymphovascular invasion, and lymph node involvement (15).

## First-line treatment and recent advances

Advanced, persistent, or recurrent CC has a 5-year survival rate of 17%. Thus, median progression-free survival (PFS) (2 to 5 months) and overall survival (OS) (5 to 16 months) is low for individuals who can’t undergo surgery or radiotherapy (16).

For many years, platinum-based chemotherapy represented the standard treatment for this patient group, with response rates of 13% and 36% for monotherapy or combination therapy, respectively (17–19). The GOG 204 study, comparing four cisplatin combinations (cisplatin/paclitaxel, cisplatin/topotecan, cisplatin/gemcitabine, and cisplatin/vinorelbine), found no difference in overall survival. While not statistically significant, a trend towards improved efficacy was observed in the cisplatin/paclitaxel group compared to the other treatment combinations. This trend was evidenced by numerically higher response rates, as well as longer progression-free survival (PFS) and overall survival (OS) (12.9 months vs. 10 months), which have established it as the preferred regimen since 2009 (17). Subsequently, the phase III JCOG0505 non-inferiority study comparing carboplatin/paclitaxel vs. cisplatin/paclitaxel demonstrated the non-inferiority of the carboplatin/paclitaxel regimen in terms of median overall survival (17.5 months vs. 18.3 months, p=0.032), with a different toxicity profile but consolidating platinum and taxane chemotherapy as the first-line treatment choice (19).

In 2014, the first targeted therapy with a benefit in metastatic, recurrent or persistent cervical cancer was established with the GOG 240 study. Bevacizumab, a monoclonal antibody, acts as an antiangiogenic agent by neutralizing vascular endothelial growth factor (VEGF), inducing tumor vascular regression, normalizing residual vasculature, and inhibiting neovascularization and, therefore, tumor growth. The combination of bevacizumab with platinum-based chemotherapy showed a 4-month overall survival advantage compared to chemotherapy alone (17 months vs. 13 months) and a response rate of 48% vs. 36% (16).

In 2021, the KEYNOTE-826 study approved the first-line immunotherapy for palliative treatment in CC. Pembrolizumab, a monoclonal antibody, binds to the PD-1 receptor, preventing its interaction with PD-L1 and PD-L2. This interference enhances the anti-tumor immune response of T cells. This phase 3, double-blind clinical trial randomized patients with persistent, recurrent, or metastatic uterine CC to receive pembrolizumab (200 mg) or placebo every 3 weeks for up to 35 cycles plus platinum-based chemotherapy and, at the investigator’s discretion, bevacizumab. In 548 patients exhibiting PD-L1 expression levels of combined positive score (CPS) ≥1%, treatment with pembrolizumab demonstrated a statistically significant improvement in both progression-free survival (PFS) and overall survival (OS) when compared to the placebo group. Specifically, the median PFS for the pembrolizumab group was 10.4 months, exceeding the 8.2 months observed in the placebo group. This difference translated to a hazard ratio (HR) of 0.62 for disease progression or death (95% confidence interval [CI], 0.50 to 0.77; p <0.001). Furthermore, the 24-month OS rate was notably higher in the pembrolizumab group, reaching 53.0% compared to 41.7% in the placebo group (HR for death, 0.64; 95% CI, 0.50 to 0.81; p <0.001). Regarding safety, the most frequently observed grade 3-5 adverse events were anemia (30.3% in the pembrolizumab group and 26.9% in the placebo group) and neutropenia (12.4% and 9.7%, respectively) (20). In 2023, the results of the study were presented, with a median follow-up of 39.1 months. In the PD-L1 ≥1% population, the administration of pembrolizumab demonstrated a notable improvement in median overall survival compared to the chemotherapy-placebo group. Specifically, the pembrolizumab group achieved a median overall survival of 28.6 months, whereas the control group reached 16.5 months (HR for death: 0.60; 95% CI: 0.49 to 0.74). This survival benefit was further accentuated in the subgroup analysis of patients with PD-L1 expression >10%. In this cohort, the median overall survival for the pembrolizumab arm was 29.6 months versus 17.4 months in the control arm (HR: 0.58; 95% CI: 0.44 to 0.78). Pembrolizumab also exhibited superiority in PFS compared to the control regimen. This was observed in both the PD-L1 CPS ≥1% population (HR: 0.58; p-value <0.0001) and the PD-L1 CPS ≥10% population (HR: 0.52; p-value <0.0001). Regarding adverse events, the incidence of grade 3 or higher events was 82.4% in the pembrolizumab group and 75.4% in the placebo group.

Recently, an exploratory subgroup analysis of this study demonstrated, in those patients with CPS ≥1%, a benefit in OS in favor of the pembrolizumab groups across all subgroups. The median OS was not reached (95% CI, 24.4-NR) in the pembrolizumab group, compared to 25.0 months (95% CI, 16.3-NR) in the placebo group among those who received bevacizumab (HR, 0.62; 95% CI, 0.45-0.87), and 17.1 months (95% CI, 14.9-20.0) in the pembrolizumab group versus 11.9 months (95% CI, 9.7-14.5) in the placebo group (HR, 0.67; 95% CI, 0.47-0.96). Regarding PFS, the HR for progression or death was significantly lower in the pembrolizumab groups compared to the placebo groups in both bevacizumab [HR of 0.61 (95% CI, 0.46-0.8)] and non-bevacizumab [HR of 0.66 (95% CI, 0.47-0.92)] subgroups. As for the use of platinum, it was shown that the median OS was 24.4 months (95% CI, 18.7-NR) in the pembrolizumab group versus 15.7 months (95% CI, 13.2-18.6) in the placebo group among those who received carboplatin (HR, 0.65; 95% CI, 0.50-0.85), and was not reached (95% CI, 22.3-NR) in the pembrolizumab group versus 24.7 months (95% CI, 16.0-NR) in the placebo group among those who received cisplatin (HR, 0.53; 95% CI, 0.27-10.04), while the PFS was 0.68 (95% CI, 0.53-0.85) in the carboplatin subgroup and 0.39 (95% CI, 0.22-0.68) in the cisplatin subgroup in CPS ≥1% (21).

## Second-line treatment options

Following initial treatment, disease progression historically presented significant challenges due to the scarcity of effective therapeutic interventions. For an extended period, a standardized second-line chemotherapy regimen remained elusive. Commonly employed chemotherapeutic agents, including taxanes, topotecan, and gemcitabine, with response rates of 13.2%, with a median PFS of 3.2 months, and a median OS of 9.3 months (22).

Until 2018, there were no promising treatments in the palliative second-line setting for patients with CC. The KEYNOTE-158 study in 2018 showed promising results with pembrolizumab. In this phase 3, double-blind study, participants were administered pembrolizumab at a dose of 200 mg every three weeks for two years, or until disease progression, unacceptable toxicity, or withdrawal by either the patient or physician. A total of 98 patients were treated, with 83.7% having PD-L1 positivity. After a median follow-up duration of 10.2 months (range: 0.6 - 22.7 months), the observed objective response rate (ORR) was 12.2% (95% confidence interval [CI]: 6.5% - 20.4%). This included three complete responses and nine partial responses, all occurring within the PD-L1-positive patient subgroup. Consequently, the ORR for PD-L1-positive patients was 14.6% (95% CI: 7.8% - 24.2%). Notably, 14.3% (95% CI: 7.4% - 24.1%) of responders within this subgroup had previously received one or more lines of chemotherapy in the recurrent or metastatic setting. Treatment-related adverse events were observed in 65.3% of the study population. The most frequently reported adverse events included hypothyroidism (10.2%), decreased appetite (9.2%), and fatigue (9.2%). Grade 3-4 treatment-related adverse events were documented in 12.2% of patients (23).

Tisotumab vedotin is a monoclonal antibody attached to a chemotherapy agent called monomethyl auristatin E (MMAE). The innovaTV 204/GOG-3023/ENGOT-cx6 study, a phase II, multicenter, open-label, single-arm study conducted in 35 centers in Europe and the United States, included 102 patients with recurrent or metastatic CC. The study enrolled patients with cervical cancer who experienced disease progression during or after bevacizumab-based chemotherapy and had undergone no more than two prior systemic treatment regimens. Participants received tisotumab vedotin at a dose of 2.0 mg/kg (maximum 200 mg) intravenously every 3 weeks until disease progression or the onset of intolerable adverse effects. The analysis included 101 patients who received at least one dose of the drug, with a median follow-up duration of 10.0 months (range: 6.1 to 13.0 months). The confirmed ORR was 24% (95% confidence interval [CI]: 16-33%), with 7% complete responses and 17% partial responses. The most frequently observed treatment-related adverse events (TRAEs) were alopecia (38%), epistaxis (30%), nausea (27%), conjunctivitis (26%), fatigue (26%), and dry eye (23%). Grade ≥3 TRAEs occurred in 28% of patients, notably including neutropenia (3%), fatigue (2%), ulcerative keratitis (2%), and various peripheral neuropathies (sensory, motor, sensorimotor, and general peripheral neuropathy). Serious TRAEs were reported in 13% of patients, with sensory-motor peripheral neuropathy (2%) and pyrexia (2%) being the most common (24).

In 2022, another treatment showed promising results in this patient group. The EMPOWER-Cervical 1 study, a phase 3 trial, in patients who had disease progression after first-line platinum-based chemotherapy, regardless of their PD-L1 status. Cemiplimab, a monoclonal antibody, similarly to pembrolizumab, targets PD-1, preventing T-cell inactivation, and enhancing T-cell mediated immune responses against tumors. This randomized controlled trial investigated the efficacy and safety of cemiplimab versus the investigator’s choice chemotherapy in women with advanced cervical cancer. A total of 608 patients were equally randomized to receive either cemiplimab (350mg every 3 weeks) or chemotherapy. The cemiplimab group demonstrated a significant improvement in median OS compared to the chemotherapy group (12.0 months vs. 8.5 months, respectively). This survival benefit was reflected in a HR for death of 0.69 (95% CI, 0.56 to 0.84; p < 0.001) favoring cemiplimab. Notably, this survival advantage remained consistent across both squamous cell carcinoma and adenocarcinoma (including adenosquamous carcinoma) histological subgroups. PFS was also significantly longer in the cemiplimab group compared to the chemotherapy group, as evidenced by a HR for disease progression or death of 0.75 (95% CI, 0.63 to 0.89; p < 0.001). The ORR was notably higher in the cemiplimab group (16.4%; 95% CI, 12.5 to 21.1) compared to the chemotherapy group (6.3%; 95% CI, 3.8 to 9.6). Interestingly, within the cemiplimab group, the response rate was 18% (95% CI, 11 to 28) for patients with PD-L1 expression ≥1% and 11% (95% CI, 4 to 25) for those with PD-L1 expression <1%. Grade ≥3 adverse events were observed in 45.0% of patients in the cemiplimab group and 53.4% of patients in the chemotherapy group. This study suggests that cemiplimab provides a significant improvement in OS and PFS compared to chemotherapy in women with advanced CC. The observed benefit was consistent across histological subgroups and PD-L1 expression levels. While adverse events were noted in both groups, the incidence of grade ≥3 events was numerically lower in the cemiplimab group (25).

**Table 1** summarizes clinical trials investigating the efficacy and safety of immunotherapy for the treatment of metastatic, recurrent, or persistent cervical cancer.

**Table 1:** Advanced, recurrent, and persistent cervical cancer targeted therapies.

Therapy	Study design	Mechanism of action	Patient population	Arms	Primary endpoint (s)	Outcomes	Adverse events
**Pembrolizumab** (20)	**KEYNOTE-826** Phase III, double-blind, randomized 1:1	Anti PD-1 antibody	Adults with advanced, recurrent, or persistent SCC, AC, or ACS of the cervix, not previously treated, ECOG 0-1.	**Experimental arm:** Pembrolizumab 200 mg IV + chemotherapy (Cisplatin 50 mg/m^2^ or Carboplatin AUC 5 + paclitaxel 175 mg/m^2^) + Bevacizumab 15 mg/m2 on day 1 per 21-day cycle **Control arm:** Placebo + chemotherapy (Cisplatin 50 mg/m^2^ or Carboplatin AUC 5 + paclitaxel 175 mg/m^2^) + Bevacizumab 15 mg/m2 on day 1 per 21-day cycle	PFS by ICR per RECIST v1.1 OS	N = 617 patients The median study follow-up duration was 39.1 months (range, 32.1-46.5 months) **All-comer** • Median PFS Pembro arm 10.4 m vs Placebo arm 8.2 m (HR 0.61, p< 0.0001) • Median OS Pembro arm 26.4 m vs Placebo arm 16.8 m (HR 0.63, p < 0.0001) **PD-L1 CPS> 1 (N= 584)** • Median PFS Pembro arm 10.5 m vs Placebo arm 8.2 m (HR 0.58, p< 0.0001) • Median OS Pembro arm 28.6 m vs Placebo arm 16.5 m (HR 0.60, p < 0.0001) **PD-L1 CPS> 1 (N= 317)** • Median PFS Pembro arm 10.4 m vs Placebo arm 8.1 m (HR 0.52, p< 0.0001) • Median OS Pembro arm 29.6 m vs Placebo arm 17.4 m (HR 0.58, p < 0.0001)	The incidence of grade ≥3 adverse events was 82.4% with pembrolizumab-chemotherapy and 75.4% with placebo-chemotherapy
**Atezolizumab** (26)	**BEATcc/ENGOT-cx10** Phase III, open-label, randomized 1:1	Anti PD-L1 antibody	Adults with advanced, recurrent, or persistent SCC, AC, or ACS of the cervix, not previously treated, ECOG 0-1.	**Experimental arm:** Atezolizumab 1200 mg + chemotherapy (Cisplatin 50 mg/m^2^ or Carboplatin AUC 5 + paclitaxel 175 mg/m^2^) + Bevacizumab 15 mg/m2 on day 1 per 21-day cycle **Control arm:** Chemotherapy (Cisplatin 50 mg/m^2^ or Carboplatin AUC 5 + paclitaxel 175 mg/m^2^) + Bevacizumab 15 mg/m2 on day 1 per 21-day cycle	PFS OS	N = 410 patients Median PFS Atezolizumab arm 13.7 m vs standard therapy 10.4 m (HR 0.62, p < 0.0001) **Interim overall survival analysis** • Median OS Atezolizumab arm 32.1 m vs standard therapy 22.8 m (HR 0.68, p < 0.0046)	Grade 3 or worse adverse events occurred in 79% of patients in the experimental group and in 75% of patients in the standard group. Grade 1–2 diarrhea, arthralgia, pyrexia, and rash were increased with atezolizumab.
**Pembrolizumab** (23)	**KEYNOTE 158** Phase II, single arm	Anti PD-1 antibody	Adults with advanced, recurrent, or persistent SCC, AC, or ACS of the cervix, that has progressed with standard-of-care systemic therapy, ECOG 0-1.	Pembrolizumab 200 mg every 3 weeks for 2 years or until progression, intolerable toxicity, or physician or patient decision	ORR	N = 98 patients Median follow-up: 10.2 months ORR was 12.2% ( 3 CR, 9 PR). All 12 responses were in PD-L1–positive tumors Median duration of response was not reached (range, 3.7 to 18.6 months)	Grade 3 3 to 4 adverse events occurred in 12.2% of patients.
**Tisoumab Vedotin** (24)	**InnovaTV 204/GOG-3023/ENGOT-cx** Phase II, open-label, single arm	Antibody drug conjugate targeting tissue factor, with tubulin inhibitor	Adults with advanced, recurrent, or persistent SCC, AC, or ACS of the cervix, who had up to 2 prior systemic treatments, including platinum-based chemotherapy +/- bevacizumab, ECOG 0-1.	Tisotumab vedotin monotherapy 2 mg/kg IV Q3w	ORR by IRC per RECIST v1.1	N = 101 patients Median follow-up at the time of analysis was 10.0 months. ORR 24% (CR 7%, PR 17%) DCR 72%	Grade 3 or worse treatment-related adverse events were reported in 28 (28%) patients Neutropenia (3% Fatigue (2%) Ulcerative keratitis (2%) Peripheral neuropathy
**Cemiplimab** (25)	**EMPOWER-CERVICAL 1/GOG-3016/ENGOT-cx9** Phase III open-label, randomized 1:1	Anti PD-1 antibody	Adults with advanced, recurrent, or persistent SCC, AC, or ACS of the cervix, not previously treated, progression after platinum/paclitaxel +/- bevacizumab chemotherapy, ECOG 0-1.	**Experimental arm:** Cemiplimab 350 mg every 21 days for 96 weeks **Control arm:** Chemotherapy of the investigator's choice (pemetrexed, topotecan, gemcitabine or vinorelbine)	OS	N = 608 patients **All patients** • Median OS Cemiplimab arm 12 m vs Chemotherapy arm 8.5 m (HR 0.69, p<0.001) **Squamous-cell carcinoma** • Median PFS Cemiplimab arm 11.1 m vs Chemotherapy arm 8.8 m (HR 0.73, p<0.006) **Adenocarcinoma or Adenosquamous Carcinoma** • Median PFS Cemiplimab arm 13.3 m vs Chemotherapy arm 7 m (HR 0.56)	Grade 3 or higher adverse events occurred in 45.0% of the patients who received cemiplimab and in 53.4% of those who received chemotherapy Anemia G3 (Cemiplimab 12 vs Chemotherapy 26%)
**Nivolumab** (27)	**CheckMate 358** Phase I/II, open-label	Anti PD-1 antibody	Adults with advanced, recurrent, or persistent SCC of the cervix or vaginal/vulvar cancer, ECOG 0-1, < 2 prior systemic therapies.	Nivolumab 240 mg every two weeks for 2 years, disease progression, or unacceptable toxicity	ORR per RECIST v1.1	N = 24 patients **N = 19 patients with cervical cáncer** ORR 26.3% (CR 15%, PR 10.5%) DOR not reached Median overall survival was 21.9 months (95% CI, 15.1 months to not reached)	Any-grade treatment-related adverse events were reported in 12 of 19 patients (63.2%) in the cervical cohort and EAS G3 (15.8%) Diarrhea Pneumonitis Liver damage)
**Balstilimab** (28)	Phase II open-label	Anti PD-1 antibody	Adults with advanced, recurrent, or persistent SCC, AC, or ACS of the cervix, whose disease relapsed after a first-line platinum-based treatment regimen, ECOG 0-1.	Bastilimab 3 mg/kg every 2 weeks for 24 months or until disease progression, intolerable toxicity	ORR per RECIST v1.1	N = 161 patients ORR: 15% ( CR 5 patients, PR 16 patients) DOR: 15.4 m **ORR rate by PD-L1 status** • Positive (n = 85) : ORR 20.0% • Negative (n = 38): ORR 7.9 % • Unknown (n = 17): ORR 5.9% **Objective response rate by histology** • Squamous (n = 85) ORR 17.6% • Adenocarcinoma (n = 48): ORR 12.5%	The most common treatment-related AEs (TRAEs) of any grade were asthenia (23%), diarrhea (12.4%), pruritus (11.8%), and fatigue (10.6%). Grade 3≥ was 11.8%, Immune-mediated enterocolitis the most frequently reported at 3.1% (five patients)
**Tisotumab Vedotin + pembrolizumab** (29)	**InnovaTV 205/GOG-3024/ENGOT-cx8** Phase Ib/II dose-expansion arms **Arm D:** TV in combination with carboplatin (1L) **Arm E:** TV with pembrolizumab (1L) **Arm F:** TV with pembrolizumab (2L/3L)	Antibody drug conjugate targeting tissue factor, with tubulin inhibitor + Anti PD-1 antibody	Adults with recurrent or stage IVB SCC, AC, or ACS of the cervix, and measurable disease at baseline per RECIST v1.1.	**Arm D:** TV 2 mg/kg on day 1 once every 3 weeks + carboplatinV¡ AUC 5 on day 1 once every 3 weeks **Arm E:** TV 2 mg/kg on day 1 once every 3 weeks, pembrolizumab 200 mg on day 1 once every 3 weeks **Arm F:** TV 2 mg/kg on day 1 once every 3 weeks, pembrolizumab 200 mg on day 1 once every 3 weeks	ORR	N = 142 patients **Arm D:** ORR 54.5% (n/N, 18/33; 95% CI, 36.4 to 71.9) DCR: 8.6 months **Arm E:** ORR , 40.6% (n/N, 13/32; 95% CI, 23.7 to 59.4) DCR: not reached **Arm F:** ORR 35.3% (12/34; 19.7 to 53.5) DCR:14.1 months	Grade ≥3 adverse events (≥15%) **Arm D:** anemia, diarrhea, nausea, and thrombocytopenia **Arm E:** Anemia **Arm F:** Anemia
**Nivolumab + Ipilimumab** (30)	**CheckMate358** Phase I/II Open-label Multiple cohort Randomized	Anti-PD-1 antibody and an anti-CTLA-4 antibody	Adults with advanced, recurrent, or persistent SCC of the cervix, ≤ 2 prior systemic therapies, ECOG 0-1, HPV status positive or unknown.	**Arm A** Nivolumab 240 mg q2w **Arm B** Nivolumab 3 mg/kg q2w + Ipilimumab 1 mg/kg q6w **Arm C** Nivolumab 1mg/kg + Ipilimumab 3 mg/kg q3w x 4 cycles, followed by nivolumab 240 mg q2w	Investigator-assessed ORR by RECIST 1.1	**Arm A:** n = 19 - First line: 5 (26%; 9 – 51) - Second or later line: 4 (27%; 8 – 55) **Arm B** n = 45 - First line: 7 (39%; 17 – 64) - Second or later line: 7 (26%; 11 – 46) **Arm C** - First line: 12 (48%; 28 – 69) - Second or later line: 6 (30%; 12 – 54)	**Arm A** Any G3-4: 4 (21%) Lead to discontinuation G3-4: 1 (5%) Treatment related SAEs G3-4: 3 (16%). **Arm B** Any G3-4: 13 (29%) Lead to discontinuation G3-4: 4 (8%) Treatment related SAEs G3-4: 8 (18%). **Arm C** Any G3-4: 52 (46%) Lead to discontinuation G3-4: 21 (19%) Treatment related SAEs G3-4: 34 (30%).

AC, adenocarcinoma; ASC, adenosquamous carcinoma; CR, complete response; CTLA-4, Cytotoxic T-lymphocyte-associated protein 4; DCR, disease control rate; IRC, independent review committee; ORR, objective response rate; OS, overall survival; PD-1, programmed cell death-1; PD-L1, programmed cell death ligand-1; PFS, Progression Free Survival RECIST, Q2W, Every 2 weeks; Q6W, Every 6 weeks; Response Evaluation Criteria in Solid Tumors; SAEs, severe adverse effects; SCC, squamous cell carcinoma; TV, Tisotumab vedotin.

Currently, there are several phase I-II clinical trials evaluating the use of immunotherapy as second-line treatment for recurrent and persistent metastatic cervical cancer, with promising outcomes expected for this patient group (**Table 2**). Furthermore, the related mechanisms of combined immunotherapy with other treatments such as chemotherapy or targeted therapies, as well as combinations of immunotherapies, are being assessed in different clinical studies and are anticipated to alter current treatment guidelines in the future.

**Table 2:** Therapies under investigation for the treatment of advanced, persistent, and recurrent cervical cancer.

Therapy	Study design	Mechanism of action	Patient population	Arms	Primary endpoint(s)
**Cadonilimab** (31)	**AK104-201-AU** Phase II Open-label Single Arm	Anti-PD-1/ CTLA-4 bispecific antibody	Adults with advanced, recurrent or persistent SCC, AC, or ACS of the cervix, who have received more than two prior systemic therapies.	Cadonilimab 6 mg/kg IV Q2W	ORR
**Geptanolimab** (32)	**Gxplore-008** Phase II Open-label Single Arm	Anti-PD-1 antibody	Adults with advanced, recurrent, or persistent cervical cancer, positive for PD-L1, who experienced disease progression or recurrence after platinum-based chemotherapy.	Geptanolimab 3 mg/kg infusion Q2W	ORR
**Camrelizumab + Famitinib** (33)	**SHR-1210-II-217** Phase II Open-label Multiple arms Randomized	Anti–PD-1 antibody and a small molecule rTKI	Adults with advanced, recurrent, or persistent SCC, AC, or ACS of the cervix, who relapsed following a platinum-based chemotherapy regimen.	**Arm 1:** Camrelizumab IV Q3W + famitinib orally once daily **Arm 2:** Camrelizumab IV Q3W **Arm 3:** Investigator’s choice of: albumin-bound paclitaxel, pemetrexed, or gemcitabine	PFS per RECIST v1.1 of Arm 1 vs Arm 2 OS of Arm 1 vs Arm 3
**Zimberelimab** (34)	**YH-S001-05** Phase II Open-label Single arm	Anti-PD-1 antibody	Adults with PD-L1 positive advanced, recurrent, or persistent cervical cancer, who progressed after at least one line of chemotherapy.	Zimberelimab monotherapy 240 mg Q2W	ORR by IRC per RECIST v1.1
**Balstilimab + Zalifrelimab** (35)	**RaPiDS** Phase II Blinded Noncomparative Randomized 1:1	Anti-PD-1 antibody and an anti-CTLA-4 antibody	Adults with advanced, recurrent, or persistent SCC, AC, or ACS of the cervix, who did not respond to a prior platinum-based chemotherapy regimen.	Balstilimab 300 mg Q3W + placebo Balstilimab 300 mg Q3W + zalifrelimab 1 mg/kg Q6W	ORR by IRC per RECIST v1.1
**Tiragolumab + Atezolizumab** (36)	**SKYSCRAPER04** Phase II Open-label Parallel-cohort Randomized 3:1	Anti-TIGIT Antibody and an anti-PD-L1 antibody	Adults with advanced, recurrent, or persistent SCC, AC, or ACS of the cervix, who progressed after 1–2 prior systemic chemotherapy regimens.	Tiragolumab 600 mg IV Q3W + atezolizumab 1200 mg IV Q3W Atezolizumab 1200 mg IV Q3W	ORR by IRC
**Sintilimab + IBI-310** (37)	**CIBI310E201** Phase II Double-blind Parallel-cohort Randomized	Anti–PD-1 antibody and an anti-CTLA-4 antibody	Adults with advanced, recurrent, or persistent cervical cancer who relapsed after a platinum-based chemotherapy regimen.	Sintilimab 200 mg + placebo Sintilimab 200 mg + IBI-310	ORR by IRC per RECIST v1.1
**Prolgolimab** (38)	**CAESURA** Phase II, Open label Single arm	Anti-PD-1 antibody	Adults with newly advanced, recurrent, or persistent SCC, AC, or ACS of the cervix.	Prolgolimab 3 mg/kg IV Q3W + chemotherapy (cisplatin or carboplatin + paclitaxel) + bevacizumab	ORR per RECIST v1.1 and iRECIST criteria

PD-1, Programmed cell death protein 1; CTLA-4, Cytotoxic T-lymphocyte-associated protein 4; SCC, Squamous Cell Carcinoma; AC, Adenocarcinoma; ACS, Adenosquamous Carcinoma; IV, Intravenous; Q2W, Every 2 weeks; ORR, Objective Response Rate; PD-L1, Programmed Death-Ligand 1; rTKI, Receptor Tyrosine Kinase Inhibitor; PFS, Progression-Free Survival; RECIST, Response Evaluation Criteria in Solid Tumors; OS, Overall Survival; IRC, Independent Review Committee; Q6W, Every 6 weeks; TIGIT, T cell immunoreceptor with Ig and ITIM domains.

## Conclusions

Cervical cancer patients facing metastatic, persistent, or recurrent disease experience a dismal prognosis, with a 5-year survival rate below 20%. This underscores the critical need for novel therapeutic interventions to improve outcomes for this patient population. Recent advancements in understanding the mechanisms of immunosuppression within the tumor microenvironment have paved the way for the development of innovative immunotherapeutic strategies. These approaches aim to counteract immunosuppressive pathways and bolster effector immune cell function, leading to promising improvements in both progression-free survival (PFS) and overall survival (OS), particularly in the first-line treatment setting.

## Recommendations

- The use of pembrolizumab with platinum + paclitaxel ± bevacizumab is recommended for patients with metastatic, recurrent, or persistent CC with PD-L1 CPS ≥1%, of squamous, adenocarcinoma, or adenosquamous histology, as first-line treatment. Quality of evidence (GRADE: High) Level of recommendation IA.
- The use of platinum + paclitaxel ± bevacizumab is recommended for patients with metastatic, recurrent, or persistent CC with PD-L1 CPS <1%, of squamous, adenocarcinoma, or adenosquamous histology, as first-line treatment. Quality of evidence (GRADE: High) Level of recommendation IA.
- For patients not eligible for combination antiangiogenic or immunotherapy plus chemotherapy, chemotherapy following international guidelines is recommended. Quality of evidence (GRADE: Moderate) Level of recommendation IIB.
- The use of pembrolizumab as monotherapy is recommended in patients with metastatic, recurrent, or persistent CC, of squamous, adenocarcinoma, or adenosquamous histology, with progression on at least one line of platinum-based chemotherapy (PD-L1 CPS ≥1%, MSI-H, dMMR, TMB-H). Quality of evidence (GRADE: Moderate) Level of recommendation IIB.
- The use of tisotumab/vedontin is recommended in patients with metastatic, recurrent, or persistent CC, of squamous, adenocarcinoma, or adenosquamous histology, with progression on chemotherapy, as second-line treatment. Quality of evidence (GRADE: Moderate) Level of recommendation IIB.
- The use of cemiplimab monotherapy is recommended in patients with metastatic, recurrent, or persistent CC, of squamous, adenocarcinoma, or adenosquamous histology, with progression on chemotherapy, as second-line treatment. Quality of evidence (GRADE: High) Level of recommendation IB.
- The use of chemotherapy as monotherapy (paclitaxel, docetaxel, gemcitabine, topotecan, vinorelbine) may be an option in second-line treatment for patients with advanced, recurrent, or persistent CC, not eligible for immunotherapy. Quality of evidence (GRADE: Moderate) Level of recommendation IIB.

## Author contributions

TG-C: Conceptualization, Data curation, Investigation, Methodology, Project administration, Supervision, Validation, Writing – original draft, Writing – review & editing. EA-B: Investigation, Resources, Writing – original draft, Writing – review & editing. JC-M: Investigation, Writing – original draft, Writing – review & editing. LC-P: Project administration, Writing – original draft, Writing – review & editing. EV-C: Writing – original draft, Writing – review & editing. RV-V: Writing – original draft, Writing – review & editing. JG-P: Data curation, Investigation, Visualization, Writing – original draft, Writing – review & editing. PC-E: Conceptualization, Supervision, Validation, Writing – original draft, Writing – review & editing.
